# Mental health status among non-medical college students returning to school during the COVID-19 pandemic in Zhanjiang city: A cross-sectional study

**DOI:** 10.3389/fpsyg.2022.1035458

**Published:** 2023-01-11

**Authors:** Xiaojun Deng, Huiting Zhang

**Affiliations:** ^1^Department of Preschool Education, Zhanjiang Preschool Education College, Zhanjiang, China; ^2^Department of Neurology, Affiliated Hospital of Guangdong Medical University, Zhanjiang, China

**Keywords:** COVID-19 pandemic, non-medical students, mental health, insomnia, anxiety, depression, Zhanjiang city

## Abstract

The coronavirus disease-2019 (COVID-19) pandemic has brought huge and continuous damage to mental health. The mental health of non-medical college students after returning to school remains largely unknown and the influencing factors were awaited to be deciphered. This cross-sectional study was launched among 1,083 non-medical students in Zhanjiang city by means of online survey (WeChat App) from August 1st, 2022 to August 7th, 2022. Knowledge about COVID-19 and attitude toward COVID-19 were assessed by using 7-items and 5-items questionnaires, respectively. Sleep quality, anxiety and depression symptoms were evaluated by Pittsburgh sleep quality index (PSQI), Hamilton depression rating scale-17 (HDRS-17) and self-rating anxiety scale (SAS), respectively. The results showed that more than half of the participants were knowledgeable about COVID-19. The majority of the participants held positive attitude toward COVID-19. The data demonstrated that 6.8% students had poor sleep quality, and 1.86, 0.37 and 0.37% students had mild, moderate and severe anxiety, respectively. About 26.7, 4.7 and 1.7% students had minimal, mild–moderate and severe depression. Female students showed higher proportions of anxiety (*p* = 0.02) and depression (*p* < 0.0001) than male students. Students with monthly household income below 3,000 RMB were more vulnerable to anxiety (*p* = 0.017) and depression (*p* = 0.004). Correlation analysis and Multivariate logistic regression analysis results showed that lower grade was positively related with anxiety and depression. Female students, income lower than 3,000 RMB/month, poor knowledge about COVID-19 and negative attitude toward COVID-19 were associated with insomnia, anxiety and depression. This study indicated that during the COVID-19 pandemic, a majority of non-medical students returning to school remained good sleep quality and a small number of students suffered from depression and anxiety. To our knowledge, this is a novel study revealing the mental health of non-medical college students concerning COVID-19 in Zhanjiang.

## Introduction

2019 coronavirus disease (COVID-19) was first reported in Wuhan, China in December 2019. COVID-19 was an infectious disease which mainly transmitted through respiratory droplets and direct contact. As of October 15th, 2021, a total of more than 239.4 million person diagnosed with COVID-19 and 4.8 million died of this life-threatening infectious disease (Zheng et al., 2022). Apart from the negative influences of COVID-19 pandemic on physical health, it also causes continuous impacts on mental health including depression and anxiety among individuals (Cao et al., 2020; Wang et al., 2020).

Several retrospective studies have suggested that infection was closely related with subsequent mood deterioration, including depression (Gunaratne et al., 2013; Rahim et al., 2022; Shokri et al., 2022). College students appear to be particularly susceptible to experiencing mental disorder and their mental state is a key concern during the pandemic (Wang X. et al., 2020). With social restriction during COVID-19, factors like decreased physical activity, prolonged sedentary behavior and poor dietary behaviors might put mental health at greater risk (Rahim et al., 2022; Sheedy O’Sullivan et al., 2022). Mental health problems would influence students’ academic performance, overall quality of life, increase suicidal ideation and suicidal behavior (Gao et al., 2022; Zhao et al., 2022). It is of great importance to pay attention to the mental health of college students. Increased incidence of mental health problems experienced by college students have been shown in various countries, including China, France, Iran and America (Lin et al., 2020; Husky et al., 2021; Oh et al., 2021; Abdolkarimi et al., 2022). In the early stage of the COVID-19 outbreak, there are 15.4–24.9% Chinese university students suffered from anxiety (Cao et al., 2020; Wang and Zhao, 2020). Another study revealed that about two-thirds of the 549 medical students experienced anxiety, depression, insomnia and distress (Essangri et al., 2021). Mental illness secondary to the infectious diseases was associated with the restriction of social activities, feelings of loneliness and inferior knowledge about the diseases (Wang et al., 2020b, 2021). After 2 years of suffering from COVID-19 epidemic, most cities in China and other countries have achieved great success in controlling COVID-19 and many students were allowed to return to school. To be note, severe acute respiratory syndrome (SARS) outbreak in 2003 and other previous pandemics have been shown to have long-term effects on mental health (Zhuo et al., 2021). The current COVID-19 pandemic might also have persistent psychological impact on college students. Several studies have devoted attention to the changes in college students’ mental health before and amidst the pandemic, while the findings yielded from these studies were controversial (Elmer et al., 2020; Wang et al., 2020b; Amendola et al., 2021). These preliminary studies motivated us to further investigate the mental health of college students under the control of the pandemic. Non-medical college students have lower understanding of COVID-19 than medical staff and medical students, making them more vulnerable to mental health issues (Lasheras et al., 2020). The mental health status among non-medical college students after returning to school remains largely unclear and the influencing factors were awaited to be explored. Large-scale studies are needed to better understand the impact of COVID-19 and associated factors on the mental health of non-medical college students, and to develop effective intervention strategies.

The aim of this study was to evaluate the mental health status among non-medical college students returning to school during the COVID-19 pandemic in Zhanjiang city. Hopefully, this study could contribute to understanding the mental health of university students in other areas of the world and might provide foundation for the strategies to protect the mental health of non-medical college students from COVID-19 damage.

## Materials and methods

### Sample size calculation

The sample size was calculated using Power Analysis and Sample Size (PASS) 11.0. The calculation formula was as follows:


$$n={\left(\frac{u_{\alpha /2}}{sin^{-1}\delta /\sqrt{p\left(1-p\right)}}\right)}^{2}$$

The PASS operating parameters were as follows: “Proportions” --- “one proportion” --- “Confidence Intervals for one proportion.” With reference to previous studies, the prevalence of anxiety in college students was 23.8% (Naser et al., 2020), we defined Proportion (*P*) = 0.238. The other parameters were set as follows: confidence level (1-*α*) = 0.95, confidence interval width = 0.1, and confidence interval was calculated by Exact (Clopper-Pearson) method and the interval type was “two-sided.” Results showed that at least 297 people were required to meet statistical differences. Considering the 10% drop-out rate, we should at least enroll 327 students. In this study, we totally enrolled 1,083 students and the number met the requirements of the trial.

### Study population and enrollment criteria

This cross-sectional study was conducted through a comprehensive social media WeChat (called Weixin in China; Tencent Inc.) from August 1st, 2022 to August 7th, 2022 (Ye et al., 2022). WeChat is the most frequently used APP in China and has been widely applied to collect mental health data (Jiang et al., 2021). Participants entered the survey by scanning a quick response (QR) code presented in the WeChat, which directed them to a survey website hosted by WenJuanXing (Changsha Haoxing Information Technology Co., Ltd., China). The inclusion criteria were as follows: non-medical college students aged 18–25 years old and voluntarily participate in this study. Exclusion criteria were as follows: students with no capacity or visual impairment or history of mental illness. The validity of the questionnaires for each participant was limited to 1 week as longer period might affect the psychological condition of the participants. The study was complied with the criteria of Strengthening the Reporting of Observation Studies in Epidemiology (STROBE), and was in accordance with the ethical standards of the institutional and/or national research committee and with the 1964 Helsinki declaration and its later amendments or comparable ethical standards. The ethics committee of the Affiliated Hospital of Guangdong Medical University approved and supervised this study (Human Investigation Committee PJKT2022-072). This study was registered in the Chinese clinical trial center, and the trial registration number is ChiCTR2200062191.^1^

Demographic, knowledge about COVID-19, attitude toward COVID-19, mental health status including insomnia, depression and anxiety were collected from all participants.

### Knowledge about COVID-19

Knowledge of COVID-19 was assessed by 7 items developed using the World Health Organization’s COVID-19 advice for the public (Wang et al., 2020b). The contents were as follows: (1) the symptoms after contracting COVID-19, (2) the signs indicating seeking health care immediately, (3) the outcomes caused by COVID-19, (4) transmission routes, (5) prevention strategies, (6) quarantine period, (7) availability of specific drug or vaccine. For each item, 0 indicated an incorrect answer and 1 indicated a correct answer, and the total score ranged from 0 to 7. A higher score suggested better knowledge of COVID-19. Poor, moderate and good knowledge of COVID-19 was defined as scores of 0–3, 4–5, and 6–7, respectively. In this study, the Cronbach’s alpha for the scale was 0.73.

### Attitude to the COVID-19 pandemic

A 5-items questionnaire was applied to measure the attitude toward the COVID-19 pandemic among college students as previously recommended with minor modification (Wang et al., 2020b). The contents were as follows: (1) being confident of knowing how to protect yourself from COVID-19, (2) do not worry about contracting COVID-19, (3) do not worry about loved ones/friends contracting COVID-19, (4) feeling hopeful to prevent COVID-19, (5) being confident that the COVID-19 will finish soon. For each item, 0 indicated disagree and 1 indicated agree, and the total score ranged from 0 to 5. A higher score indicated a more positive attitude toward COVID-19 prevention and control. Scores of 0–2, 3, and 4–5 were regarded as negative, neutral and positive attitude toward COVID-19, respectively. The Cronbach’s alpha for the scale in this study was 0.82.

### Insomnia, depression and anxiety evaluation

Insomnia was assessed by Pittsburgh sleep quality index (PSQI). Depression and anxiety were evaluated by Hamilton depression rating scale-17 (HDRS-17) and self-rating anxiety scale (SAS), respectively. These scales were considered as valuable screening instruments for insomnia, depression and anxiety in different populations (Huang et al., 2018; Liu et al., 2020; Han et al., 2021). On the basis of previous studies and considering that the study was conducted on Chinese students, Chinese version of these scales were applied in this study (Jiang et al., 2021). In furtherance, the demographics information including age, gender, grade and household income were collected.

As for PSQI, there were 19 questions in this scale with seven components: duration of sleep, sleep disturbance, sleep latency, subjective sleep quality, sleep efficiency, daytime dysfunction and sleep medicine intake. The score for each component is 3 points and the total PSQI scale is 21 points. Higher score implied worse sleep quality. PSQI score > 5 and ≤ 5 were considered as poor and good sleep quality, respectively (Benítez et al., 2022). Cronbach’s *α* was 0.83 for this scale. The contents of HDRS-17 included depressed mood, feelings of guilt, suicide, insomnia (early in the night/middle of the night/early hours of the morning), work and activities, retardation, agitation, anxiety psychic, anxiety somatic, somatic symptoms gastro-intestinal, general somatic symptoms, genital symptoms, hypochondriasis, loss of weight and insight. HDRS-17 score < 8 indicated no depression (Huang et al., 2018), score 8–16 indicated minimal depression, score 17–24 indicated mild–moderate depression (Sampaio-Junior et al., 2018), score ≥ 25 indicated severe depression (Versiani et al., 2005). Cronbach’s α was 0.86 for this scale. SAS was applied to assess anxiety. The SAS questionnaire consist of 20 questions, and each question ranged from 1 to 4 according to the frequency of symptoms. The score for each item was calculated to obtain the raw score, and the standard score was calculated *via* the raw score multiplied by 1.25. Anxiety was classified into four different levels: normal (≤49), mild (50–59), moderate (60–70) and severe anxiety (≥70) (Liu et al., 2020). Cronbach’s *α* was 0.84 for this scale.

The informed consent of each study population was obtained and the answers to the specific demographic questions and insomnia, depression and anxiety scales were collected electronically. The questionnaires mentioned above all showed good reliability and validity in the surveys among college students (Huang et al., 2018; Raniti et al., 2018; Wang and Zhao, 2020; Wang et al., 2020a).

### Statistical analysis

Statistical analysis was performed by using GraphPad Prism 7.0 and SPSS 22.0 (SPSS Inc., Chicago IL, United States). D’Agostino-Pearson omnibus test was used to determine normal distribution. Statistical description was expressed as median ± inter-quartile range (IQR) for the variables with non-normal distribution. The chi-square test or Fisher’s exact test was used to compare anxiety and depression among different gender and household income. Spearman’s analysis was conducted to examine the correlations between insomnia, anxiety, depression and related parameters like gender, age, income, knowledge about COVID-19 and attitude toward COVID-19. The significant factors were included in the stepwise multivariate logistic regression analysis to study their association with insomnia, anxiety and depression (Wang et al., 2022). Raw Cronbach’s αs for total and subscale scores and raw corrected item-total, item-component, and component-total Spearman’s rho (*r*_s_) correlations were calculated to assess the questionnaires’ internal consistency. Cronbach’s *α* ≥ 0.70 and corrected correlations (*r*_s_) ≥ 0.30 were recognized to have adequate internal consistency (Spira et al., 2012). Results were expressed as adjusted odds ratios (OR) with the corresponding 95% confidence intervals (95%*CI*). Differences with *p* < 0.05 were considered statistically significant.

## Results

### Characteristics of the participants

**Figure 1:**
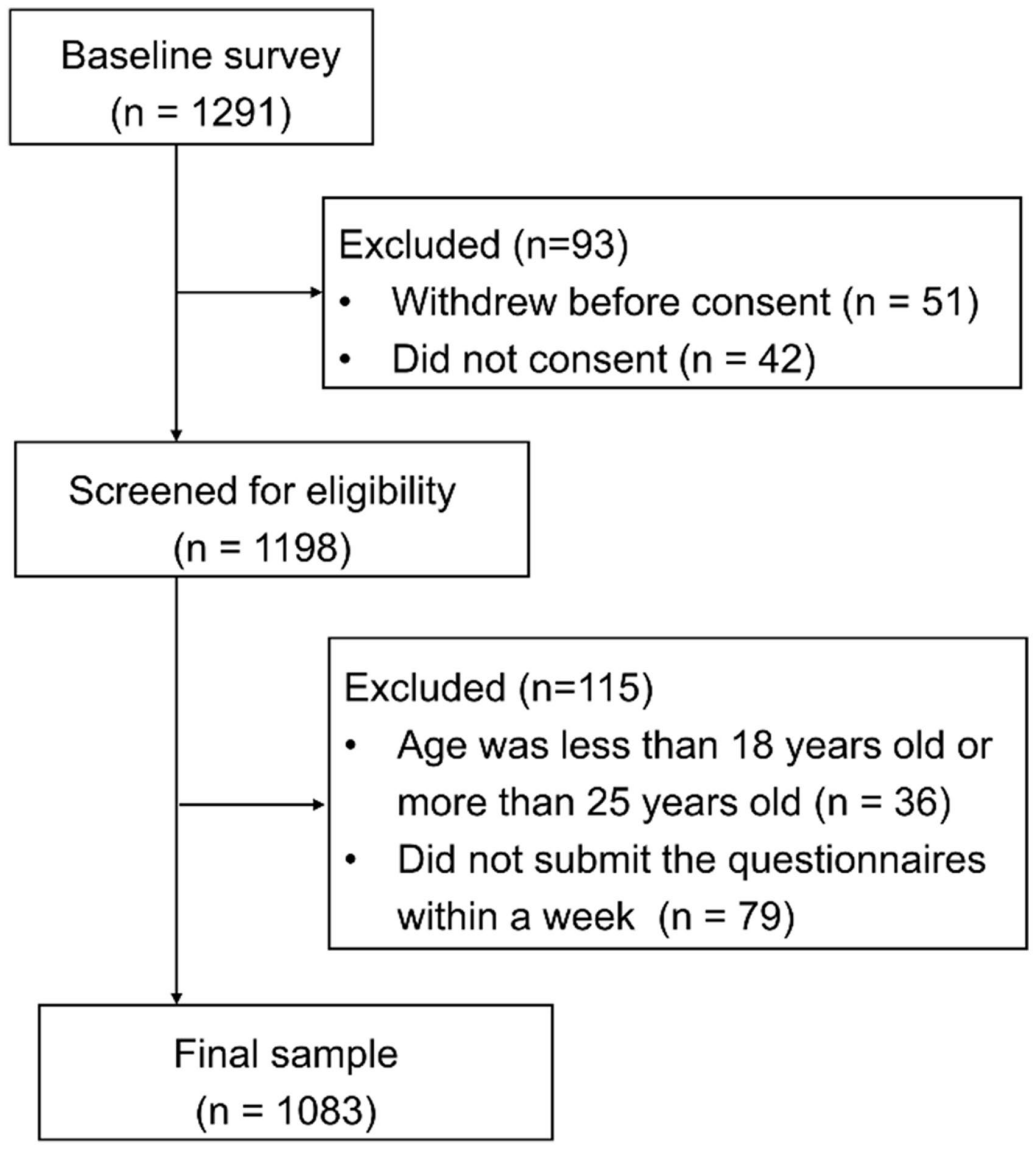
Flowchart diagram of study population.

A total of 1,083 non-medical students were enrolled in this study (Figure 1). As shown in Table 1, the majority of participants were females (*n* = 681, 62.9%). There were 475 participants (43.9%) aged between 18 and 19 years old and 608 participants (56.1%) aged between 20 and 25 years old. Around half of the participants were freshman (*n* = 525, 48.5%), and the rest were sophomore (42.6%) and junior or above (8.9%). There were 294 participants (27.1%) owned a monthly household income of less than 3,000 RMB and 789 participants (72.9%) had more than 3,000 RMB each month.

**Table 1:** Demographics of the participants.

Demographics	*n* (%)
Age	
18–19 years old	475 (43.9%)
20–25 years old	608 (56.1%)
Gender	
Male	402 (37.1%)
Female	681 (62.9%)
Grade	
Freshman	525 (48.5%)
Sophomore	462 (42.6%)
Junior or above	96 (8.9%)
Household income	
<3,000/month	294 (27.1%)
≥3,000/month	789 (72.9%)

### Knowledge about COVID-19

As shown in Table 2, most individuals had a correct understanding of the COVID-19. The correct answers for the 7 items of COVID-19 knowledge ranged from 65.2–77.6%. There were 66.8% (723/1083) and 70.4% (762/1083) students had good knowledge about the symptoms after contracting COVID-19 and the signs of indicating seeking health care immediately. More than half of the participants were familiar with the outcomes caused by COVID-19 (74.3%, 805/1083), transmission routes (74.9%, 811/1083) and prevention strategies of COVID-19 (77.6%, 840/1083), quarantine period (65.2%, 706/1083) and availability of specific drug or vaccine of COVID-19 (72.4%, 784/1083).

**Table 2:** Knowledge related to the COVID-19 epidemic among non-medical college students.

Variables	*n* (%)
The symptoms after contracting COVID-19	
Correct	723 (66.8%)
Incorrect	360 (33.2%)
The signs of indicating seeking health care immediately	
Correct	762 (70.4%)
Incorrect	321 (29.6%)
The outcomes caused by COVID-19	
Correct	805 (74.3%)
Incorrect	278 (25.7%)
Transmission routes	
Correct	811 (74.9%)
Incorrect	272 (25.1%)
Prevention strategies on COVID-19	
Correct	840 (77.6%)
Incorrect	243 (22.4%)
Quarantine period	
Correct	706 (65.2%)
Incorrect	377 (34.8%)
Availability of specific drug or vaccine	
Correct	784 (72.4%)
Incorrect	299 (27.6%)

### Attitude toward COVID-19

A majority of individuals reported being confident in knowing how to protect themselves from COVID-19 (96.1%, 1041/1083), being confident that the COVID-19 will end soon (93.4%, 1011/1083), feeling hopeful to prevent COVID-19 (91.6%, 992/1083). There were 49.7 and 39.9% participants worried about loved ones/friends and themselves contracting COVID-19, respectively (Table 3).

**Table 3:** Attitude toward to the COVID-19 epidemic among non-medical college students.

Variables	*n* (%)
Being confident of knowing how to protect yourself from COVID-19	
Agree	1,041 (96.1%)
Disagree	42 (3.9%)
Do not worry about contracting COVID-19	
Agree	432 (39.9%)
Disagree	651 (60.1%)
Do not worry about loved ones/friends contracting COVID-19	
Agree	538 (49.7%)
Disagree	545 (50.3%)
Feeling hopeful to prevent COVID-19	
Agree	992 (91.6%)
Disagree	91 (8.4%)
Being confident that the COVID-19 will finish soon	
Agree	1,011 (93.4%)
Disagree	72 (6.6%)

### Prevalence of sleep quality, anxiety and depression among the participants

As presented in Table 4, most participants had good sleep quality (93.2%, 1010/1083), and very few students (6.8%, 73/1083) suffered from insomnia. The prevalence of anxiety among the participants were as follows: no anxiety (97.4%, 1055/1083), mild anxiety (1.86%, 20/1083), moderate anxiety (0.37%, 4/1083) and severe anxiety (0.37%, 4/1083). The proportions of no, minimal, mild–moderate and severe depression were 66.9, 26.7, 4.7 and 1.7%, respectively.

**Table 4:** Prevalence of insomnia, anxiety, and depression among the participants.

Variables	*n* (%)	Median ± IQR	*p* value
Sleep quality			
Good	1,010 (93.2%)	3 ± 2	<0.0001^***^
Poor	73 (6.8%)	6 ± 2	
Anxiety degree			
No anxiety	1,055 (97.4%)	27 ± 7.5	<0.0001^***^
Anxiety	28 (2.6%)	64.3 ± 3.8	
Mild anxiety	20 (1.86%)	56 ± 4	
Moderate anxiety	4 (0.37%)	65 ± 4.35	
Severe anxiety	4 (0.37%)	72 ± 3	
Depression degree			
No depression	725 (66.9%)	3 ± 3	<0.0001^***^
Depression	358 (33.1%)	28.5 ± 3.75	
Minimal depression	289 (26.7%)	10 ± 4	
Mild–moderate depression	51 (4.7%)	19 ± 3.75	
Severe depression	18 (1.7%)	28 ± 3.5	

^***^p < 0.001.

### Association between insomnia, anxiety, depression and related factors

**Figure 2:**
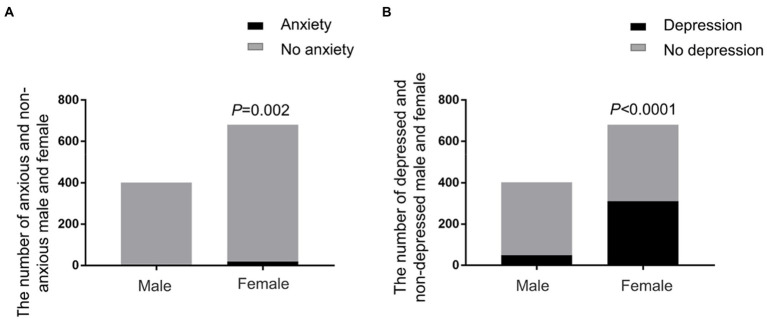
Anxiety and depression of students of different genders. **(A)** The prevalence of anxiety in male and female students. Female students showed significantly higher proportion of anxiety than male students (Fisher’s exact test, *p* = 0.02). **(B)** The prevalence of depression in male and female students. Female students represented more depressive emotion than male students (Chi-square test, *p* < 0.0001).

**Figure 3:**
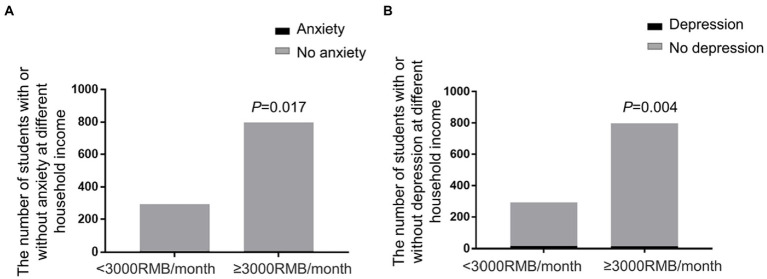
Anxiety and depression of students of different household income. **(A)** The prevalence of anxiety in different household income. The students with household income below 3,000 RMB/month showed significantly higher anxiety (Fisher’s exact test, *p* = 0.017). **(B)** The prevalence of depression in different household income. The students with household income below 3,000 RMB/month had higher rate of depression (Fisher’s exact test, *p* = 0.004).

Female students obviously represented with more anxiety and depression than male students (anxiety: Fisher’s exact test, *p* = 0.02, Figure 2A; depression: Chi-square test, *p* < 0.0001, Figure 2B). Subjects with household income below 3,000 RMB per month showed significantly higher anxiety and depression than those with monthly income above 3,000 RMB (anxiety: Fisher’s exact test, *p* = 0.017, Figure 3A; depression: Fisher’s exact test, *p* = 0.004, Figure 3B).

Correlation analysis results showed that female and low monthly income were positively associated with insomnia (female: *r* = 0.17, *p* < 0.001; income: *r* = −0.11, *p* < 0.001, Table 5). Grades were related with anxiety and depression (anxiety: *r* = −0.092, *p* = 0.02; depression: *r* = −0.14, *p* < 0.001, Tables 6, 7). Good knowledge about COVID-19 and positive attitude toward COVID-19 were negatively associated with insomnia, anxiety and depression (Tables 5–7). Age was not correlated with insomnia, anxiety and depression (Tables 5–7).

**Table 5:** Correlation between insomnia and related parameters.

Variables	Age	Gender (female)	Grade	Income	Knowledge about COVID-19	Attitude toward COVID-19
*r* value	−0.03	0.17	0.02	−0.11	−0.15	−0.13
*p* value	0.35	<0.001^***^	0.49	<0.001^***^	<0.001^***^	<0.001^***^

^***^p < 0.001.

**Table 6:** Correlation between anxiety and related parameters.

Variables	Age	Gender	Grade	Income	Knowledge about COVID-19	Attitude toward COVID-19
*r* value	0.05	0.021	−0.09	0.026	−0.12	−0.254
*p* value	0.11	0.49	0.002^**^	0.39	<0.001^***^	<0.001^***^

^**^p < 0.01, ^***^p < 0.001.

**Table 7:** Correlation between depression and related parameters.

Variables	Age	Gender	Grade	Income	Knowledge about COVID-19	Attitude toward COVID-19
*r* value	0.03	0.013	−0.14	0.017	−0.19	−0.22
*p* value	0.32	0.66	<0.001^***^	0.57	<0.001^***^	<0.001^***^

^***^p < 0.001.

We further performed multivariate logistic regression analysis to explore the risk factors associated with insomnia, anxiety and depression. Gender, grade, income, knowledge about COVID-19 and attitude toward COVID-19 were included in the multiple logistic regression analysis. As the data shown in Table 8, female (OR = 0.02, 95%CI: 0.008–0.061), household income lower than 3,000 RMB per month (OR = 13.78, 95%CI: 7.459–25.45), poor knowledge about COVID-19 (OR = 0.82, 95%CI: 0.728–0.915) and negative attitude toward COVID-19 (OR = 0.69, 95%CI: 0.538–0.872) were positively associated with the presence of insomnia. Female (OR = 4.02, 95%CI: 1.216–10.834), low income (OR = 4.08, 95%CI: 1.412–12.081), poor knowledge about COVID-19 (OR = 0.48, 95%CI: 0.370–0.612) and negative attitude toward COVID-19 (OR = 0.52, 95%CI: 0.347–0.788) were positively correlated with the presence of anxiety. Female (OR = 1.08, 95%CI: 1.015–1.161), freshman (OR = 1.87, 95%CI: 1.074–3.266), low monthly income (OR = 2.76, 95%CI: 1.506–5.473), poor knowledge about COVID-19 (OR = 0.85, 95%CI: 0.795–0.898) and negative attitude toward COVID-19 (OR = 0.79, 95%CI: 0.703–0.898) were positively associated with the presence of depression.

**Table 8:** Multivariate logistic regression among insomnia, anxiety, depression, and risk factors.

Independent variables	Insomnia		Anxiety		Depression	
	OR (95% CI)	*p*	OR (95% CI)	*p*	OR (95% CI)	*p*
Gender (female)	0.02 (0.008–0.061)	<0.001^***^	4.02 (1.216–10.834)	0.047^*^	1.08 (1.015–1.161)	0.016^*^
Grade (freshman)	0.78 (0.260–2.333)	0.656	1.07 (0.673–1.703) -	0.775	1.87 (1.074–3.266)	0.027^*^
Income (<3000RMB/month)	13.78 (7.459–25.45)	<0.001^***^	4.08 (1.412–12.081)	0.014^*^	2.76 (1.506–5.473)	0.003^**^
Knowledge about COVID-19	0.82 (0.728–0.915)	<0.001^***^	0.48 (0.370–0.612)	<0.001^***^	0.85 (0.795–0.898)	<0.001^***^
Attitude toward COVID-19	0.69 (0.538–0.872)	0.002^**^	0.52 (0.347–0.788)	<0.002^**^	0.79 (0.703–0.898)	<0.001^***^

^*^p < 0.05, ^**^p < 0.01, ^***^p < 0.001.

## Discussion

This study investigated the COVID-19 pandemic on mental health of 1,083 non-medical college students returning to school in Zhanjiang, China. The research found that most students acquired good knowledge about COVID-19 and held positive attitude toward COVID-19. There were 6.8% students suffered from insomnia, 2.6% students had mild–severe anxiety, and approximately 33.1% students had different degrees of depression. Female students, household income lower than 3,000 RMB per month, poor knowledge about COVID-19 and negative attitude toward COVID-19 were associated with insomnia, anxiety and depression, and freshmen were more vulnerable to depression.

COVID-19 seemed to have adverse impact on the long-term consequences of mental health (Zhuo et al., 2021). A national mental health study conducted in February and April 2020 among Chinese adolescents showed that the prevalence of depression and anxiety increased over time evidently (Chen et al., 2021). The prevalence of depressive and anxiety symptoms among back-to-school children was elevated in comparison to that during the home quarantine period (Xie et al., 2022). The prevalence of insomnia, anxiety and depression among high school students after reopening schools during the COVID-19 was 42.7 to 63.4% (Puteikis et al., 2022). A survey of 5,285 adults in the United States demonstrated that the incidence of adverse mental health symptoms during the later phase of the COVID-19 pandemic (September 2020) was higher than that in June 2020 (Czeisler et al., 2021). Some studies depicted that mental health of college students deteriorated in the few months following the COVID-19 pandemic (Lipson et al., 2022; Shiratori et al., 2022; von Keyserlingk et al., 2022). Some other evidence indicated a decrease in anxiety symptoms with time among university students (Amendola et al., 2021), and some data shown that college students just experienced minor changes during the pandemic outbreak (Pieh et al., 2021; Stamatis et al., 2022). These inconsistent findings highlighted the need for sustained attention to the dynamics of college students’ mental health across time and space. Our study focused on the psychological symptoms in non-medical college students who have experienced long-term home quarantine and have now back to school. During the prevalence of COVID-19, the students accepted online learning instead of face-to-face classes, and the activities of students in public places were restricted (Qin et al., 2021; Regmi and Lwin, 2021). We suggested that these altered lifestyle and social patterns might contribute to the development of mental illness (Wang et al., 2021; Zhuo et al., 2021). Mental disorder could lead to reduced quality of life and higher healthcare burden (Rodrigues et al., 2015). Therefore, providing early recognition of mental illness and support for the vulnerable students is a vital need. It is reported that the incidence of depression among general university students was 12.2–56.8% (Wang et al., 2020; Yu et al., 2021), and approximately 22.4–35.5% medical students represented with depressive symptoms during the COVID-19 pandemic (Xiao et al., 2020; Xie et al., 2020). The prevalence of depression among non-medical students in this study (33.1%) seemed to have no difference from previously published data on medical students and was lower than that of returning high school students. Previous studies pointed out that the incidence of anxiety among general university students and medical students were 7.7–15.43% (Wang et al., 2020; Wang and Zhao, 2020) and 22.1–28% (Lasheras et al., 2020; Xiao et al., 2020), respectively. A recent meta-analysis revealed that the prevalence of anxiety in general population during the COVID-19 pandemic was 31.9% (Salari et al., 2020). The prevalence of anxiety and insomnia among back-to-Wuhan university students was 44 and 37.5%, respectively (Wu et al., 2022). In this study, the incidence of anxiety (2.6%) was lower than previously reported in general university students and medical students, and was particularly lower than the data reported in the general population and the university students returning to school. Besides, a majority of non-medical students showed good sleep quality in this study. The mental health improvements shown in this study might be due to the emphasis placed by schools and government on enhancing COVID-19 knowledge among college students. For the general population, they obtained information from a variety of sources and probably lacked the ability to distinguish information accurately (Xiong et al., 2021). Poor COVID-19 knowledge was correlated with negative emotions like tension and depression (Lee et al., 2021). In addition, optimistic perception and familiarity with COVID-19 might help control the rapid spread of COVID-19 and decrease the incidence of mental illness (Zhong et al., 2020; Yin et al., 2022). Our data revealed that most participants had good knowledge about COVID-19 and held positive attitude toward COVID-19, with the exception of a minority of students who were concerned about contracting COVID-19 for themselves and their loved ones/friends. Our results indicate that the non-medical schools and government of Zhanjiang provide adequate COVID-19 knowledge and implement stringent policies promptly during the pandemic. Positive mental health attitudes can be improved by the popularization of mental health literacy, public awareness of COVID-19 prevention and control, and social support (Zhong et al., 2020).

In addition to evaluating the influences of participants’ knowledge about COVID-19 and attitude toward COVID-19 on their mental health, our study also investigated other potential factors for the development of depression, anxiety and insomnia. One research revealed that students in higher grades had lower prevalence of sleep disturbance compared with those in the lower grades (Li et al., 2020). The sleep quality among different grades in this study did not show statistical difference, indicating that the freshmen enrolled were adaptable to the new circumstances during the COVID-19 pandemic. Age was not correlated with insomnia, anxiety and depression in this study, probably owning to the students enrolled were very similar in age. We found that female students were more prone to sleep disorder, anxiety and depression. The results were in accordance with the previous study demonstrating that women were approximately twice as likely to experience mood disturbance as men, including sleep problems, anxiety and depression (Mong and Cusmano, 2016; Serpytis et al., 2018). This sexual discrepancy might result from different sex steroids levels. For example, testosterone has been regarded as an important sex steroid to resist anxiety and depression (Zarrouf et al., 2009). In furtherance, the influence of economic status of the participants on anxiety and depression should be noted. It is demonstrated that the diabetic patients of higher-income showed a significant decline for anxiety (Al-Ayed et al., 2021). People with low socioeconomic status had high incidences of anxiety and depression (Linder et al., 2020). Similarly, our result also revealed that subjects with higher household incomes had lower rates of anxiety and depression.

To the best of our knowledge, this is the first and largest study that explore the prevalence of depression, anxiety and insomnia among non-medical college students returning to school during COVID-19 pandemic in Zhanjiang city. Our study can provide information on risk factors associated with psychological effects in the countries such as China where the pandemic is under control. The results of the study might be helpful for future research in other countries. There are a few limitations that should be acknowledged. Given the use of an online survey, there might exist some response bias. Though our data are in line with many previous studies, it is hard to establish a causal relationship between pandemic context and mental health disorders in a cross-sectional study. The study might be improved in the future by increasing the correlation analysis between mental health and other factors such as perceived social support, living environment (urban or rural area), physical activity, sedentary time, and dietary behaviors (Ivbijaro et al., 2021; Shibata et al., 2021; Rahim et al., 2022; Silva et al., 2022; Zhao et al., 2022). Furthermore, mood disturbance has been reported to be associated with inflammatory processes (Beurel et al., 2020). We suggest that it would be interesting to study the immunological changes of the participants in the next study.

## Conclusion

During the COVID-19 prevalence, non-medical college students returning to school in Zhanjiang experienced no higher levels of anxiety and depression, and most students represented with good sleep quality. The results from this study might help in guiding healthcare practitioners and policymakers to work out appropriate and feasible interventions to recognize and treat the students with mental disorders. Most importantly, universities may be able to apply these results to build mental health profiles of students, which could help identify more ‘at risk’ students in the face of COVID-19.

## Data availability statement

The original contributions presented in the study are included in the article/supplementary material, further inquiries can be directed to the corresponding author.

## Ethics statement

The studies involving human participants were reviewed and approved by the ethics committee of the Affiliated Hospital of Guangdong Medical University. The patients/participants provided their written informed consent to participate in this study.

## Author contributions

XD and HZ designed the study. XD developed the questionnaire, recruited the participants, and wrote the main manuscript text. HZ revised the manuscript, analyzed the data, and had full access to all the data in the study and final responsibility for the decision to submit for publication. All authors reviewed and approved the final manuscript.

## Funding

This work was supported by Guangdong Medical Research Foundation, Grant/Award Number: B2018048; Science and technology research project of Zhanjiang City, Grant/Award Number: 2018B01012; Research Foundation of Guangdong Medical University, Grant/Award Number: GDMUM201807; and Scientific research project of Zhanjiang Preschool Education College (Research center for rural preschool education in Western Guangdong, Guangdong Province), Grant/Award Number: ZY2022XJZX10.

## Conflict of interest

The authors declare that the research was conducted in the absence of any commercial or financial relationships that could be construed as a potential conflict of interest.

## Publisher’s note

All claims expressed in this article are solely those of the authors and do not necessarily represent those of their affiliated organizations, or those of the publisher, the editors and the reviewers. Any product that may be evaluated in this article, or claim that may be made by its manufacturer, is not guaranteed or endorsed by the publisher.

## Footnotes

^1^ https://www.chictr.org.cn/
